# *In vivo *validation of the adequacy calculator for continuous renal replacement therapies

**DOI:** 10.1186/cc3517

**Published:** 2005-04-07

**Authors:** Zaccaria Ricci, Gabriella Salvatori, Monica Bonello, Tirak Pisitkun, Irene Bolgan, Giuseppe D'Amico, Maurizio Dan, Pasquale Piccinni, Claudio Ronco

**Affiliations:** 1 Consultant, Department of Intensive Care, Policlinico Umberto I, Rome, Italy; 2 Research fellow, Department of Nephrology, St Bortolo Hospital, Vicenza, Italy; 3 Specialist registrar, Department of Nephrology, St Bortolo Hospital, Vicenza, Italy; 4 Research fellow, Department of Nephrology, St Bortolo Hospital, Vicenza, Italy; 5 Statistician, Department of Nephrology, St Bortolo Hospital, Vicenza, Italy; 6 Research fellow, Department of Intensive Care, Policlinico Umberto I, Rome, Italy; 7 Head, Department of Intensive Care, St Bortolo Hospital, Vicenza, Italy; 8 Head, Department of Nephrology, St Bortolo Hospital, Vicenza, Italy

## Abstract

**Introduction:**

The study was conducted to validate *in vivo *the Adequacy Calculator, a Microsoft Excel-based program, designed to assess the prescription and delivery of renal replacement therapy in the critical care setting.

**Methods:**

The design was a prospective cohort study, set in two intensive care units of teaching hospitals. The participants were 30 consecutive critically ill patients with acute renal failure treated with 106 continuous renal replacement therapies (CRRT). Urea clearance computation was performed with the Adequacy Calculator (*K*_CALC_). Simultaneous blood and effluent urea samples were collected to measure the effectively delivered urea clearance (*K*_DEL_) at the beginning of each treatment and, during 73 treatments, between the 18th and 24th treatment hour. The correlation between 179 computed and 179 measured clearances was assessed. Fractional clearances for urea were calculated as sp*Kt*/*V *(where sp represents single pool, *K *is clearance, *t *is time, and *V *is urea volume of distribution) obtained from software prescription and compared with the delivered sp*Kt*/*V *obtained from empirical data.

**Results:**

We found that the value of clearance predicted by the calculator was strongly correlated with the value obtained from computation on blood and dialysate determination (*r *= 0.97) during the first 24 treatment hours, regardless of the renal replacement modality used. The delivered sp*Kt*/*V *(1.25) was less than prescribed (1.4) from the Adequacy Calculator by 10.7%, owing to therapy downtime.

**Conclusion:**

The Adequacy Calculator is a simple tool for prescribing CRRT and for predicting the delivered dose. The calculator might be a helpful tool for standardizing therapy and for comparing disparate treatments, making it possible to perform large multi-centre studies on CRRT.

## Introduction

Acute renal failure (ARF), as a component of the multiple organ failure syndrome, affects morbidity and mortality in critically ill patients [1]. This is still the case even though several aspects of medical care and applied technology in ARF were improved. Much development in renal replacement therapy (RRT) is ongoing, concerning new techniques, new membranes, and new integrated equipment. However, it is still unclear whether a correlation between treatment dose and outcome exists and no consensus has been reached on how much treatment is adequate [2,3]. A long-term, large-scale, multi-center study to determine how the outcome of critically ill patients is affected by RRT dose and modality (intermittent or continuous, diffusive or convective) is still lacking [4,5]. This is in part due to the complexity of data collection and to the variety of existing standards in RRT prescription and dose evaluation.

We tested a computer program called 'Adequacy Calculator for ARF', a simple and manageable tool designed to prescribe RRT dose and to collect information about the quantity of delivered treatments. Pisitkun and colleagues [6] have described this Microsoft excel based program and its algorithms in a previous paper. Once the required parameters are entered, it calculates urea clearance and fractional clearance, sp*Kt*/*V *(sp = single pool; *K *= clearance, *t *= time, *V *= urea volume of distribution) for each continuous RRT (CRRT) modality.

## Materials and methods

We prospectively collected data from 106 consecutive continuous renal replacement treatments administered to 30 patients with acute renal failure in the intensive care unit of St Bortolo Hospital and Policlinico Umberto I in the period from March 2003 to November 2004. The decision to start and to withdraw RRT, anticoagulation and prescription of net ultrafiltration rate were left to institutional protocols (Table 1). Treatments were delivered at different modalities and machine settings depending on the preference of the prescribing physician, but a final sp*Kt*/*V *of 1.4 had to be prescribed by means of the Adequacy Calculator. The plasma filtration fraction, in the case of postfilter reinfusion of replacement solution, was kept below 20%. By protocol, filters were changed after 24 hours of treatment, or earlier if clotting occurred. The available membranes were Diacap α (1.2 m^2^, polysulphone, B Braun) and Aquamax HF 12 (1.2 m^2^; polyethersulphone; Edwards Lifescience) for 59 and 47 treatments respectively. Sixty-four treatments were performed with bicarbonate-buffered replacement and dialysate fluids, and 42 with lactate-buffered fluids. Daily operative treatment times and downtimes were reported. Intermittent treatments were excluded from the analysis.

The Adequacy Calculator estimated urea clearance (*K*_CALC_) for each different modality and machine setting (Additional file 1). The calculator estimation is founded on the assumption that urea sieving coefficient is equal to unity for convective therapies; at the same time the calculator assumes that complete saturation of spent dialysate occurs under continuous veno-venous hemodialysis (CVVHD) conditions.

To correlate *K*_CALC _with effectively delivered instantaneous urea clearance (*K*_DEL_), simultaneous samples from prefilter blood and effluent were collected during each treatment, to measure urea concentration; 106 blood and 106 effluent samples were withdrawn during the first hour from the start of RRT; 73 blood-effluent samples were withdrawn between the 18th and 24th hours (in 33 cases treatment was stopped before the 18th hour). *K*_DEL _was calculated as described in Additional file 1. One hundred and six *K*_DEL _values at treatment start (*T*_0_) and 73 values after 18 to 24 hours of treatment (*T*_18_) were correlated with 106 and 73 *K*_CALC _values obtained for the same treatments.

**Figure 1:**
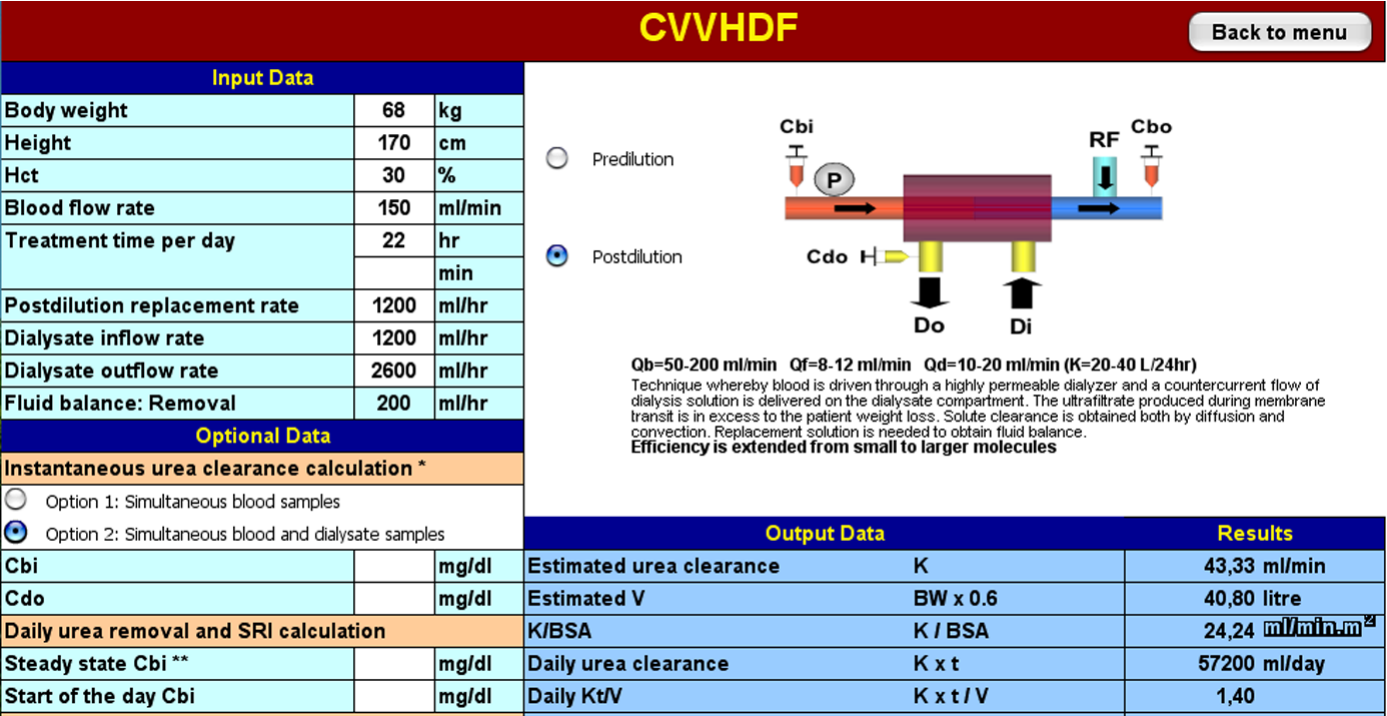
An Adequacy Calculator worksheet: continuous veno-venous hemodiafiltration (CVVHDF) is delivered in a 70 kg patient. Post-dilution mode is selected, machine settings and prescribed treatment time per day are entered on the upper left panel: estimated urea clearance (*K*_CALC_) and 'daily *Kt*/*V*' (sp*Kt*/*V*_CALC_) are displayed on the right. In the lower left panel it is possible to obtain *K*_DEL _measure after entering prefilter blood (Cbi) and effluent (Cdo) urea concentration: in this case, when operative times are entered, 'daily *Kt*/*V*' cell displays effectively delivered fractional clearance (sp*Kt*/*V*_DEL_).

The calculator prescribed sp*Kt*/*V*_CALC _after *K*_CALC_, the expected treatment time and patient's body weight (for assessment of urea volume of distribution, *V*; Fig. 1) had been entered. sp*Kt*/*V*_DEL _was calculated from *K*_DEL_, *V *and effective operative treatment times (Additional file 1).

### Statistical analysis

Statistical analysis was performed with the SPSS 11.5 software package. Data are reported as means ± standard deviation (SD). Correlations between estimated and measured urea clearance were performed with the Pearson correlation coefficients (*r*). sp*Kt*/*V*, *K*_CALC _and *K*_DEL _have no normal distribution, so we used a Mann-Whitney test (between two samples) or a Kruskal-Wallis test (between three or more samples) to indicate whether groups had different locations. *P *< 0.05 was considered statistically significant.

## Results

A total of 106 RRTs administered to 30 patients were analysed with the Adequacy Calculator. An average of 3.5 treatment days was examined for each patient. Nineteen post-dilution continuous veno-venous hemofiltrations (CVVHs), 23 pre-dilution CVVHs, 23 CVVHDs and 41 post-dilution continuous veno-venous hemodiafiltrations (CVVHDF) were prescribed. The duration of each treatment was 17 ± 6 hours. The daily operative treatment time was 20 ± 3 hours with a downtime of 3 ± 2 hours. Thirty-three treatments lasted less than 18 hours (16 CVVH and 17 CVVHD); 73 treatments lasted more than 18 hours (26 CVVH, 6 CVVHD and 41 CVVHDF). Examined clearances ranged from 15 ml min^-1 ^to 100 ml min^-1 ^(Table 2), this wide range being explained by variability in patients' weights and prescribed treatment times: because the prescribed sp*Kt*/*V*_CALC _was maintained at a constant 1.4, a 35 kg patient treated for 24 hours with a *K*_CALC _of 20 ml min^-1 ^obtained the same fractional clearance as a 98 kg patient dialyzed for 12 hours with a *K*_CALC _of 100 ml min^-1^.

**Figure 2:**
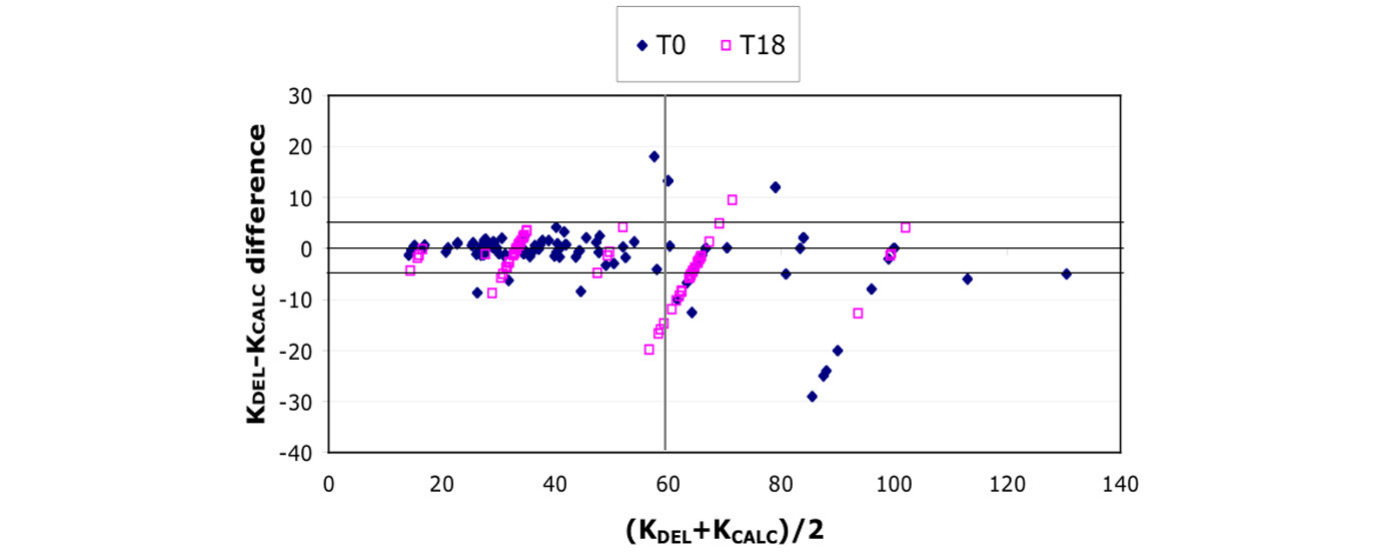
Bland–Altman correlation between urea clearance obtained by two methods: urea clearance calculated with the described software (*K*_CALC_) and urea clearance obtained by direct measure on prefilter blood and effluent samples (*K*_DEL_). It is possible to distinguish the correlations between *K*_CALC _and *K*_DEL_, at therapy start (*T*_0_) and after 18 to 24 hours of uninterrupted therapy (*T*_18_).

The difference between *K*_DEL _and *K*_CALC _was -1.75 ± 5.9 ml min^-1^. Applying a Pearson correlation we obtained *r *= 0.97; a significant (*P *= 0.022) decrease in calculator accuracy in predicting effectively delivered clearance was obtained when data from the *K*_CALC _< 60 ml min^-1 ^subgroup (*r *= 0.95) were compared with data from the *K*_CALC _> 60 ml min^-1 ^subgroup (*r *= 0.89). A Bland-Altman analysis (Fig. 2) confirmed high correlation: this result was particularly evident up to an average clearance ([*K*_DEL _+ *K*_CALC_]/2) of 60 ml min^-1^, with the *K*_DEL _- *K*_CALC _difference never exceeding a standard deviation of 5.9 ml min^-1^, whereas for [*K*_DEL _+ *K*_CALC_]/2 > 60 ml min^-1^, the *K*_DEL _- *K*_CALC _difference tended to increase. However, we found that 155 of 179 (87%) *K*_DEL _values fell within a ± 15% *K*_CALC _error: in 5 cases the calculator underestimated, and in 19 overestimated, the delivered clearance. No significant *K*_DEL _- *K*_CALC _difference was observed when *T*_0 _and *T*_18 _clearances were analysed (*P *= 0.54) and no significant difference (*P *= 0.394) was observed when *K*_CALC _> 60 ml min^-1 ^in the *T*_0 _subgroup and *K*_CALC _> 60 ml min^-1 ^in the *T*_18 _subgroup were analyzed: calculator accuracy was not affected by filter lifespan (Table 3).

**Figure 3:**
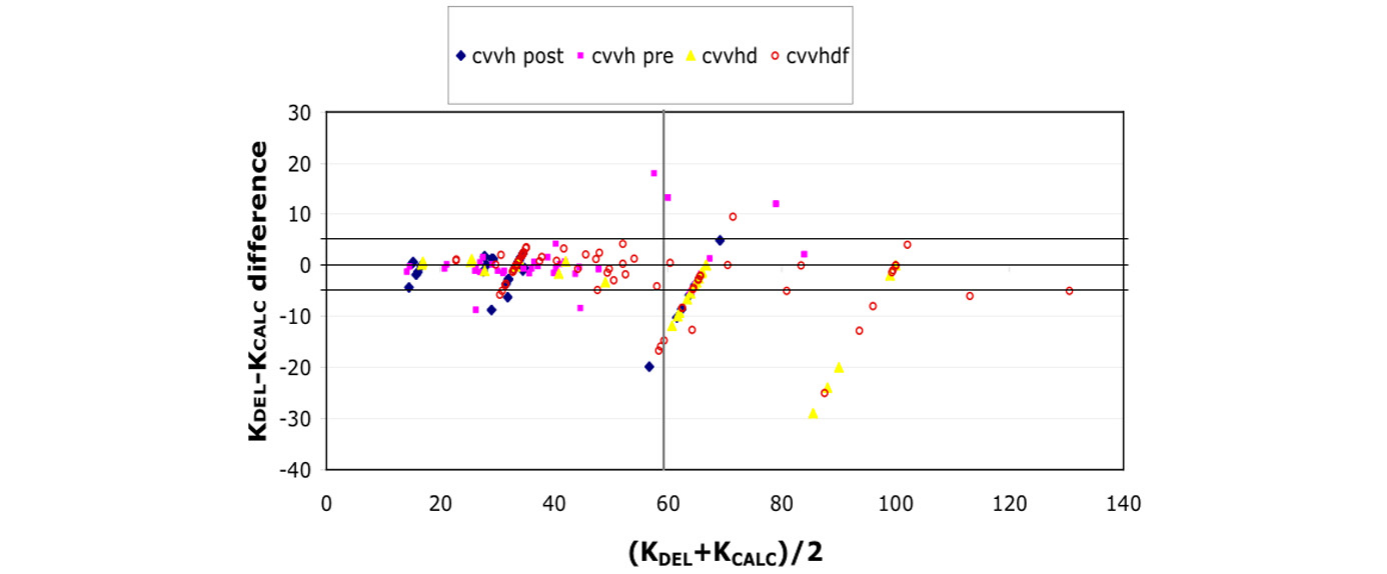
Bland–Altman analysis. The same data as above are used; here it is possible to distinguish between different modalities. Parallel lines indicate standard deviation. CVVH, continuous veno-venous hemofiltration; CVVHD, continuous veno-venous hemodialysis; CVVHDF, continuous veno-venous hemodiafiltration; *K*_CALC_, calculator-estimated urea clearance; *K*_DEL_, delivered clearance evaluated from urea concentrations on simultaneous blood and effluent samples.

After analysis of each modality group, correlations were still high: *r*_CVVHpre _= 0.96, *r*_CVVHpost _= 0.96, *r*_CVVHD _= 0.97 and *r*_CVVHDF _= 0.98 (Fig. 3), with no significant difference between groups (*P *= 0.099) (Table 3).

Membrane type did not affect the *K*_DEL _- *K*_CALC _correlation: r obtained for Diacap M and Aquamax HF 12 were 0.96 and 0.97 respectively (*P *= 0.1).

The average sp*Kt*/*V*_DEL _obtained during our treatments was 1.25 ± 0.6; the delivered/prescribed ratio was 0.89 (Table 3); the delivered fractional clearance was significantly less than the prescribed sp*Kt*/*V*_CALC _of 1.4 (*P *= 0.045).

## Discussion

Ideal marker molecules and performance parameters to compare treatment dose in different techniques are difficult to establish. In spite of its moderate toxicity, urea is currently used as a marker of RRT adequacy because it is easily measurable and, representing the end of protein metabolism, its accumulation during kidney failure defines the requirement for dialysis while its elimination defines the efficiency of treatment. Because urea is equally distributed at steady state in body water compartments, its volume of distribution (*V*) equals total body water. Urea is therefore a surrogate of the low-molecular-mass toxins. In chronic hemodialysis, the treatment dose of RRT is defined as a fractional clearance, *Kt*/*V*, where *K *is the instantaneous clearance, *t *is treatment time and *V *is the volume of distribution of the marker molecule. This is a dimensionless parameter that represents the *efficacy *of treatments, and allows comparison between different therapies and among different patients. In fact, different instantaneous clearances, representing treatment *efficiency*, can yield comparable results in terms of efficacy only if correlated with treatment time and the patient's total body water. A *Kt*/*V *value of 1.2 is an established maker of adequacy that has been shown to be correlated with morbidity and mortality in patients with end-stage kidney disease [7-11]. *Kt*/*V *has not yet been validated as a marker of adequacy in patients with acute renal failure, but it seems that a good rationale exists for its use in continuous therapies. Theoretically, in its original conception, clearance was thought to evaluate renal function of disparate individuals whose kidneys were operating 24 hours per day and blood levels were at steady state. Similarly, after some days of CRRT, patients' urea levels approach a real steady state (never obtained with intermittent dialysis) and post-dialysis rebound is not present. It is thus reasonable to consider urea distribution volume as in a single-pool kinetic model (sp*Kt*/*V*).

Recently, Brause and colleagues [12] stated that sp*Kt*/*V *is a valuable tool for evaluating continuous hemofiltration, and higher values (0.8 versus 0.53) were correlated to improve uremia control and acid–base balance. Ronco and colleagues [2] showed an improved outcome with postdilution hemofiltration delivered at 35 ml h^-1 ^kg^-1 ^in a 450-patient population. Setting a sp*Kt*/*V *threshold that could guide clinicians towards adequate treatments, we should possibly meet the target of 35 ml h^-1 ^kg^-1^, which, delivered as a 24-hour treatment, may translate into a sp*Kt*/*V *of 1.4 independently of the RRT modality.

We found that the Adequacy Calculator was able to predict the delivered urea clearance accurately, regardless of which CRRT modality was selected; the correlation between prediction and effective delivery remained high over a time range of 24 hours. When clearances above 60 ml min^-1 ^were prescribed, the calculator showed a tendency to overestimate effective clearances: this overestimation remained generally within an error of 15%.

Considering our results and the dissociation between treatment delivery and calculator estimation when high clearances are involved, as could occur with low-efficiency extended dialysis or high-volume hemofiltration, a slight correction to prevent the overestimation of effective treatment delivery is strongly advised. Nevertheless, even in the presence of an error of up to 15%, which is unlikely to occur, the delivered *Kt*/*V *in 24 hours will always approach the target value of 1.2.

The use of the calculator allowed us to strictly monitor our treatments during the study period and described an average 10.7% (*P *< 0.05) decrease in delivery of therapy in comparison with prescribed dose. The differences between prescribed and delivered dose in critically ill patients with ARF undergoing intermittent hemodialysis were analyzed by Evanson and colleagues [13]; they found that only 30% of dialysis delivered a *Kt*/*V *of 1.2; high patient weight, male sex and low blood flow were the limiting factors affecting RRT administration. In our population, this decrease in delivery was sometimes due to overestimation of *K*_CALC _by the calculator, and, more often, to operative treatment time, which was often shorter than the prescribed treatment time (during bag substitution and filter change the treatment was not administered). Our observation is consistent with a recent large retrospective analysis [14]. In this setting, when a 'standardized' downtime is foreseen, treatment prescription might be adjusted to correct for the time of zero clearance.

However, all these considerations must be seen in the light of an absolute lack of any previous attempt to adjust treatment dose to specific target levels. Furthermore, a clear understanding of adequate levels of renal replacement therapy has yet to be achieved. In this state of absence of information and of wide ignorance of the field, the calculator might have the merit of placing the issue of treatment dose among the priorities of critical care nephrology: a dose prescription should be made before embarking on an extracorporeal blood purification technique, and the delivered treatment dose should be monitored.

The limitations of this study are as follows. A subgroup analysis of net ultrafiltration (UF) prescription, daily treatment length and downtime difference within different modalities was not performed: in our opinion these factors do not affect Adequacy Calculator accuracy. Slight subgroup disparities in *K*_CALC _prescription within different modalities were present because prescribing physicians were not asked to modify their usually preferred modality. The effect of different blood pump flow rates on error in *K*_CALC _was not evaluated: higher blood flow rates might have decreased some *K*_CALC _- *K*_DEL _differences, especially when high-volume treatments were used. The observational nature of our study did not allow us to analyse all possible prescriptions systematically: a dedicated study should be performed. Finally, partial thromboplastin time, prothrombin time, platelet levels, anticoagulation and administration of drotrecogin alfa were not taken into consideration; however, our study showed that, during a period of 24 hours, urea sieving coefficient and clearance were not significantly affected by treatment duration and, indirectly, by progressive filter clogging. In our experience, anticoagulation parameters affect the lifespan of membranes in the first 24 hours but do not affect urea clearance.

## Conclusion

We assume that by using simple CRRT parameters and the Adequacy Calculator it is possible to simply prescribe and closely monitor the dose of different continuous therapies. This tool might help in future prospective studies to correlate different dose prescriptions with different clinical outcomes.

## Key messages

• 	The Adequacy Calculator is a Microsoft Excel-based program, designed to assess the prescription and delivery of renal replacement therapy in the critical care setting.

• 	A prospective study was performed in order to evaluate correlation between calculated and measured clearances.

• 	The value of clearance predicted by the calculator was strongly correlated with the value obtained from determination on blood and dialysate: the Adequacy Calculator is a reliable tool for prescribing CRRT and for predicting the delivered dose.

## Abbreviations

ARF = acute renal failure; CRRT = continuous renal replacement therapy; CVVH = continuous veno-venous hemofiltration; CVVHD = continuous veno-venous hemodialysis; CVVHDF = continuous veno-venous hemodiafiltration; *K *= clearance; *K*_CALC _= calculator-estimated urea clearance; *K*_DEL _= delivered clearance evaluated from urea concentrations on simultaneous blood and effluent samples; RRT = renal replacement therapy; sp*Kt*/*V *= single pool fractional clearance for urea;*t *= time; *V *= urea volume of distribution.

## Competing interests

The author(s) declare that they have no competing interests.

## Authors' contributions

ZR designed the study, participated in data collection and drafted the paper. MB, GS, EA and GD participated in data collection. IB provided statistical expertise. MD and PP revised the article. CR designed the study and participated in data interpretation. All authors read and approved the final manuscript.

## Supplementary Material

Additional File 1 A pdf file containing Adequacy Calculator algorithms for urea clearance and single pool fractional clearance computation is provided. Click here for file

## Figures and Tables

**Table 1:** Characteristics of patients

Parameter	Value (mean ± SD; range)
Total no. of patients	30
Age (years)	58 ± 12
Weight (kg)	73.7 ± 15.7 (35–105)
SAPS II score	38 ± 12
No. of evaluated treatments	106
Examined treatment days	3.5 ± 1.1
Prescribed sp*Kt*/*V*	1.4
Diagnosis	
Sepsis/septic shock	8
Bowel perforation	3
Aortic aneurysm repair	3
Pneumonia	5
Hemorrhagic shock	2
Pancreatitis	1
Multiple trauma	5
Cardiogenic shock	3
Urine output at RRT start (ml per 24 h)	150 ± 70
Urea levels at RRT start (mg dl^-1^)	124 ± 55
Urea levels after 24 h from RRT start (mg dl^-1^)	98 ± 50
Urea levels at RRT withold	68 ± 53
Creatinine levels at RRT start (mg dl^-1^)	2.9 ± 0.9
Creatinine levels after 24 h from RRT start (mg dl^-1^)	2.4 ± 1.25
Creatinine levels at RRT withold	1.8 ± 0.6
No. of patients receiving heparin < 500 U h^-1^	12
No. of patients receiving heparin > 500 U h^-1^	11
No. of patients receiving no anticoagulation	7

RRT, renal replacement therapy; SAPS II, Systems Approach Problem Solver II.

**Table 2:** Treatments characteristics

Parameter	Value
No. of treatments	106
Total no. of examined *K*_*CALC*_/*K*_*DEL*_(ml min^-1^)	179 (106 *T*_0_; 73 *T*_18_)
CVVH_post_	38 (19 *T*_0_; 19 *T*_18_)
CVVH_pre_	30 (23 *T*_0_; 7 *T*_18_)
CVVHD	29 (23 *T*_0_; 6 *T*_18_)
CVVHDF	82 (42 *T*_0_; 42 *T*_18_)
Total prescribed *K*_CALC _(ml min^-1^)	48.6 ± 24; 18–100
CVVH_post_	35.6 ± 16; 16–66
CVVH_pre_	48.8 ± 19; 15–83
CVVHD	56.2 ± 28; 20–100
CVVHDF^a^	52.2 ± 24; 23–100
Total prescribed net UF (ml h^-1^)	250 ± 100; 0–500
Treatment hours per day	20 ± 3; 8.5–24
Treatment hours per run	17 ± 6; 5–24
Downtime (hours)	3 ± 2

^a^During continuous veno-venous hemodiafiltration (CVVHDF) modality, prescribed clearance was delivered with even hemofiltration and hemodialysis flow rates. Where errors are given, results are means ± SD; ranges follow a semicolon. CVVH, continuous veno-venous hemofiltration; CVVHD, continuous veno-venous hemodialysis; *K*_CALC_, calculator-estimated urea clearance; *K*_DEL_, delivered clearance evaluated from urea concentrations on simultaneous blood and effluent samples; *T*_0_, at therapy start; *T*_18_, after 18 to 24 hours of uninterrupted therapy.

**Table 3:** Calculated-delivered urea clearance correlation

Parameter	*K*_CALC _- *K*_DEL _(ml min^-1^)	*r*
Total	-1.7 ± 5.9	0.97
CVVH_post_	-2 ± 4.3	0.96
CVVH_pre_	1.4 ± 6	0.96
CVVHD	-4.5 ± 7.7	0.97
CVVHDF	-1.8 ± 5.3	0.98
Subgroups		
*K*_CALC _< 60 ml min^-1^	0.05 ± 3.3	0.95
*K*_CALC _> 60 ml min^-1^	-5.2 ± 8.2^a^	0.89
*K*_CALC _at *T*_0_	-1.04 ± 6.3	0.97
*K*_CALC _at *T*_18_	-2.8 ± 5.2	0.96
*K*_CALC _< 60 ml min^-1 ^at *T*_0_	0.4 ± 3.6	0.95
*K*_CALC _> 60 ml min^-1 ^at *T*_0_	-4.8 ± 9.7^a^	0.87
*K*_CALC _< 60 ml min^-1 ^at *T*_18_	-0.6 ± 2.9	0.94
*K*_CALC _> 60 ml min^-1 ^at *T*_18_	-5.5 ± 6.5^a^	0.89

Delivered sp*Kt*/*V*	1.25 ± 0.6	
Delivered/prescribed	0.89	

^a^*P *< 0.05 (referred to total *K*_CALC _- *K*_DEL _difference).

Where errors are given, results are means ± SD. CVVH, continuous veno-venous hemofiltration; CVVHD, continuous veno-venous hemodialysis; CVVHDF, continuous veno-venous hemodiafiltration; *K*_CALC_, calculator-estimated urea clearance; *K*_DEL_, delivered clearance evaluated from urea concentrations on simultaneous blood and effluent samples; *T*_0_, therapy start; *T*_18_, 18 to 24 hours of uninterrupted therapy.

## References

[B1] Brivet F, Kleinknecht D, Loriat P, Landais P (1996). The French Study Group on Acute Renal Failure: acute renal failure in intensive care units – causes, outcome, and prognostic factors on hospital mortality: a prospective, multicenter study. Crit Care Med.

[B2] Ronco C, Bellomo R, Homel P, Brendolan A, Dan M, Piccinni P, La Greca G (2000). Effect of different doses in continuous veno-venous haemofiltration on outcomes of acute renal failure: a prospective randomised trial. Lancet.

[B3] Gotch FA (2001). Daily hemodialysis is a complex therapy with unproven benefits. Blood Purif.

[B4] Ronco C, Bellomo R (1998). Continuous renal replacement therapy: evolution in technology and current nomenclature. Kidney Int.

[B5] Clark WR, Ronco C (1998). Renal replacement therapy in acute renal failure: solute removal mechanisms and dose quantification. Kidney Int.

[B6] Pisitkun T, Tiranathanagul K, Poulin S, Bonello M, Salvatori G, D'Intini V, Ricci Z, Bellomo R, Ronco C (2004). A practical tool for determining the adequacy of renal replacement therapy in acute renal failure patients. Contrib Nephrol.

[B7] Parker T, Husni L, Huang W, Lew N, Lowrie EG (1994). Survival of hemodialysis patients in the United States is improved with a greater quantity of dialysis. Am J Kidney Dis.

[B8] NKF/DOQI (2001). Clinical practice guidelines for haemodialysis adequacy: updater 2000. Am J Kidney Dis.

[B9] Owen WF, Chertow GM, Lazarus JM, Lowrie EG (1998). Dose of hemodialysis and survival: differences by race and sex. JAMA.

[B10] Daugirdas JT, Depner TA, Gotch FA, Greene T, Keshaviah P, Levin NW, Schulman G (1997). Comparison of methods to predict equilibrated *Kt*/*V *in the HEMO pilot study. Kidney Int.

[B11] Gotch F, Sargent J (1985). A mechanistic analysis of the National Cooperative Dialysis Study (NCDS). Kidney Int.

[B12] Brause M, Neumann A, Schumacher T, Grabensee B, Heering P (2003). Effect of filtration volume of continuous venovenous hemofiltration in the treatment of patients with acute renal failure in intensive care units. Crit Care Med.

[B13] Evanson JA, Himmelfarb J, Wingard R, Knights S, Shyr Y, Schulman G, Ikizler TA, Hakim RM (1998). Prescribed versus delivered dialysis in acute renal failure patients. Am J Kidney Dis.

[B14] Venkataraman R, Kellum JA, Palevsky P (2002). Dosing patterns for CRRT at a large academic medical center in the United States. J Crit Care.

