# Supplementary material for: In vivo validation of the adequacy calculator for continuous renal replacement therapies
Source: Crit Care. 2005 Apr 7;9(3):R266–73. doi: 10.1186/cc3517 (PMC1175890; doi:10.1186/cc3517)
Supplement: Additional File 1 — A pdf file containing Adequacy Calculator algorithms for urea clearance and single pool fractional clearance computation is provided. [file cc3517-S1.pdf]

Additional file 1

CVVH POSTDILUTION

Estimated urea clearance (K_{CALC}):

K_{CALC} = Quf

Delivered urea clearance (K_{DEL}):

K_{DEL} = Cuf *Quf/Cbi

Where, Cuf = ultrafiltrate urea level (mg/dl).

Quf = ultrafiltration rate (ml/min).

Cbi = prefilter urea level (mg/dl).

CVVH PREDILUTION

Estimated urea clearance (K_{CALC}):

K_{CALC} = Quf / [1 + (Qr / Qb)]

Delivered urea clearance (K_{DEL}):

K_{DEL} = Cuf *Quf/Cbi

Where, Qr = predilution fluid replacement rate (ml/min).

Qb = blood flow rate (ml/min).

CVVHD

Estimated urea clearance (K_{CALC}):

K_{CALC} = Qdo

Delivered urea clearance (K_{DEL}):

K_{DEL} = Cdo * Qdo /Cbi

Where, Cdo = dialysate outflow urea nitrogen level (ml/min).

Qdo = dialysate outflow rate (ml/min).

Cbi = prefilter urea level (mg/dl).

CVVHDF POSTDILUTION

Estimated urea clearance (K_{CALC}):

K_{CALC} = Quf + Qdo

Delivered urea clearance (K_{DEL}):

K_{DEL} = Cuf-do * (Quf + Qdo) / Cbi

Where, Cuf-do = ultradiafiltrate outflow urea level (mg/dl).

UREA VOLUME OF DISTRIBUTION

V (L): patient's body weight (Kg) * 0.6

FRACTIONAL CLEARANCE:

$SpKt/V_{CALC}$: K_{CALC} (ml/min)*prescribed treatment time (min)/V (ml)

$SpKt/V_{DEL}$: K_{DEL} (ml/min)*operative treatment time (min)/V (ml)
